# Direct short-term effects of EBP teaching: change in knowledge, not in attitude; a cross-cultural comparison among students from European and Asian medical schools

**DOI:** 10.3402/meo.v17i0.19623

**Published:** 2012-10-31

**Authors:** Indah S. Widyahening, Geert J.M.G. van der Heijden, Foong Ming Moy, Yolanda van der Graaf, Sudigdo Sastroasmoro, Awang Bulgiba

**Affiliations:** 1 Department of Community Medicine, Faculty of Medicine, Universitas Indonesia, Jakarta, Indonesia; 2 Department of Epidemiology, Division Julius Center for Health Sciences and Primary Care, University Medical Center Utrecht, Utrecht, Netherlands; 3 Center for Clinical Epidemiology and Evidence-based Medicine, Faculty of Medicine, Universitas Indonesia – Cipto Mangunkusumo Hospital, Jakarta, Indonesia; 4 Department of Otorhinolaryngology, Division Surgical Specialisms, University Medical Center Utrecht, Utrecht, Netherlands; 5 Julius Centre University of Malaya, Department of Social and Preventive Medicine, Faculty of Medicine, University of Malaya, Kuala Lumpur, Malaysia

**Keywords:** Evidence Based Medicine, knowledge, attitude, behavior, medical students

## Abstract

**Introduction:**

We report about the direct short-term effects of a Clinical Epidemiology and Evidence-based Medicine (CE-EBM) module on the knowledge, attitude, and behavior of students in the University Medical Center Utrecht (UMCU), Universitas Indonesia (UI), and University of Malaya (UM).

**Methods:**

We used an adapted version of a 26-item validated questionnaire, including four subscales: knowledge, attitude, behavior, and future use of evidence-based practice (EBP). The four components were compared among the students in the three medical schools before the module using one-way ANOVA. At the end of the module, we measured only knowledge and attitudes. We computed Cronbach's α to assess the reliability of the responses in our population. To assess the change in knowledge and attitudes, we used the paired *t*-test in the comparison of scores before and after the module.

**Results:**

In total, 526 students (224 UI, 202 UM, and 100 UMCU) completed the questionnaires. In the three medical schools, Cronbach's α for the pre-module total score and the four subscale scores always exceeded 0.62. UMCU students achieved the highest pre-module scores in all subscales compared to UI and UM with the comparison of average (SD) score as the following: knowledge 5.04 (0.4) vs. 4.73 (0.69) and 4.24 (0.74), *p*<0.001; attitude 4.52 (0.64) vs. 3.85 (0.68) and 3.55 (0.63), *p*<0.001; behavior 2.62 (0.55) vs. 2.35 (0.71) and 2.39 (0.92), *p*=0.016; and future use of EBP 4.32 (0.59) vs. 4.08 (0.62) and 3.7 (0.71), *p*<0.01. The CE-EBM module increased the knowledge of the UMCU (from average 5.04±0.4 to 5.35±0.51; *p*<0.001) and UM students (from average 4.24±0.74 to 4.53±0.72; *p*<0.001) but not UI. The post-module scores for attitude did not change in the three medical schools.

**Conclusion:**

EBP teaching had direct short-term effects on knowledge, not on attitude. Differences in pre-module scores are most likely related to differences in the system and infrastructure of both medical schools and their curriculum.

## Introduction

Evidence-based practice (EBP) is being considered as an essential component of training in the health professions (1). An increasing number of medical schools around the world have incorporated EBP teaching into their curricula (2). As such, establishing an EBP module involves ‘a planned educational experience that encompasses behavioral goals, instructional methods, and the actual experience of the learners’ (3). Learning goals encompass three components: knowledge, skills, and attitudes (1). Changes in attitudes and perceptions as well as knowledge are important precursors to changes in behavior (2). In spite of the need, incorporation of EBP in teaching in an existing curriculum is a challenging task for some medical schools, especially those with limited resources as in developing countries. The alternative approach is to adapt an established curricullum.

An existing Clinical Epidemiology and Evidence-based Medicine (CE-EBM) module originating from a European country (University Medical Center Utrecht [UMCU], the Netherlands has been adopted and implemented in two Asian countries (Universitas Indonesia [UI] and University of Malaya [UM]). The learning goals of this module aim to produce doctors who are able to practice all five steps of EBP (asking, acquiring, appraising, applying, and assessing) or an EBP ‘doer’ as defined by Straus et al. (4). Our experience shows that adoption of an existing evidence-based medicine (EBM) curriculum is feasible (5).

As this CE-EBM module is a new addition to the curriculum in both medical schools (UI and UM), there is a need for a comprehensive evaluation of learning effects. The initial quality evaluations showed that the module was well received by the medical students and their teachers. In general, the students’ knowledge increased, learning goals of the module have been achieved, while students indicated to have learned much (5). These were achieved despite differences in the medical education structure, schools’ curriculum, healthcare system, as well as culture of the communities.

This report aims to: (a) study the direct short-term effects of the EBM module on the knowledge and attitude of the students in UMCU, UI, and UM and (b) compare the knowledge, attitude, and behavior (KAB) of students in UMCU, UI, and UM.

## Methods

### EBM module

The content and structure of the EBM module implemented in the three medical schools has been described elsewhere (5). The module was implemented first in the UMCU before it was adapted and adopted by UI and UM. In short, it follows the principles of the Sicily statement on EBM teaching (1) and includes lectures on the design and conduct of diagnostic, therapeutic, prognostic, and etiologic studies, computer practices on literature search and data analysis, tutored group discussions on EBM assignments and moderated plenary assignment presentations. Small groups of five students are assigned to develop an evidence-based case report (EBCR), that is, comprehensive best evidence summary for information needs from patient care (6). Students submit their EBCR in written form and as oral presentations at the end of the module. Students are evaluated and marked for their EBCRs, a written exam, and for their activity during small group discussions.

To fit the local situations, the module is implemented differently. In UMCU, the clinical epidemiology (CE) and EBM modules are run separately as a 6-week full time CE module targeted at 3rd year students and a 6-week part-time EBM module for 6th (final) year students. In UI and UM, both modules were merged into an integrated CE-EBM module. The CE-EBM module in UI was given to the medical students as a 4-week module (condensed) at the end of their 4th year. In UM, it was conducted dispersed within the 3 months period of social and preventive medicine (SPM) module for 3rd year medical students.

### Instrument selection

In finding the most suitable instrument for our purposes, we chose a questionnaire assessing KAB, which was developed by a team from the University of Hong Kong (7). This questionnaire is mentioned in the Sicily statement (1) and is considered relevant for the evaluation of EBM teaching and has been thoroughly validated (7). The questionnaire contains 26 items that are classified in to four subscales: knowledge (5 items), attitudes (6 items), application (6 items), and future use (9 items). Students are asked to mark each item on a 6-point Likert or adjectival scale (strongly agree – strongly disagree, not at all – completely, never – all the time, very difficult – very easy, completely useless – very useful, very unwilling – very willing). With Cronbach's α of the whole questionnaire and of each subscale exceeding 0.7, the internal consistency was shown to be satisfactory (7, 8).

### Instrument adaption

The questionnaire was originally in English and no translation to local languages (Indonesian, Malay or Dutch) was considered necessary. Based on the assessment of the fit of the items for the local situation and purposes, almost all of the questions were considered as highly relevant. However, modification was implemented to four of the attitude questions, which asked the students to compare their present attitude with that of 1 year ago. As we thought that the statements might confuse the students, we decided to only ask the respondent to score according to their present condition.

### Data collection

The questionnaire was completed by students enrolled in the CE-EBM module in the three medical schools. The questionnaire was administered twice: at the beginning of the module and at the end. At the start of the module, the complete 26-item questionnaire was completed. We did not expect changes in behavior in the short term, immediately at the end of the module. Therefore, at the end of the module, only the 11 items of the knowledge and attitude subscales were to be completed.

### Instrument consistency

To assess the internal consistency of the adapted questionnaire, Cronbach's α was computed separately for each school. The subscale scores were obtained by calculating the mean score of all the items in each subscale. One-way ANOVA was used to compare the knowledge, attitude, behavior, and future use of EBP among students in the three medical schools at the start of the module. The effect of the EBM module on the knowledge and attitude of the medical students in each school was assessed by comparing the score before and after the module using the paired *t*-test. All analyses were performed using SPSS Version 11.0 (SPSS Inc., Chicago, IL).

### Ethical consideration

The CE-EBM module implemented in UI and UM was to become part of the medical curriculum and the effect assessment become part of module evaluation, which should be undertaken by all of the students. As such, the managerial considerations on the curriculum revision dominated over possible ethical considerations. However, all data were analyzed and reported anonymously.

## Results

The questionnaire was implemented in 2010 in the UI and UM and 2010–2011 in UMCU. In total, 526 students completed the questionnaires, all in the clinical stage of their curriculum: participants were 100 students from UMCU in their 6th year, 224 students from UI in their 4th year, 202 students from UM in their 3rd year. The questionnaires completed before the start of the module at the three medical schools showed that Cronbach's α exceeded 0.74 for the total questionnaire and exceeded 0.62 for all four subscales (Table 1).

**Table 1:** Analysis of internal validity (Cronbach's α) of the pre-EBP module scores for subscales for knowledge, attitudes, application and future use of evidence-based practice (EBP) in the Universitas Indonesia (UI), University of Malaya (UM), and University Medical Center Utrecht (UMCU)

Components	UMCU (*n* =100)	UI (*n* =224)	UM (*n* =202)
EBP knowledge	0.745	0.862	0.854
Attitudes toward EBP	0.688	0.765	0.626
Personal application and use (behavior) of EBP	0.677	0.757	0.754
Future use of EBP	0.833	0.814	0.841

The pre-module scores in the three medical schools are presented in Table 2. Overall, significant differences were observed in the students’ knowledge, attitude, application, and future use of EBP in the three medical schools. Compared to students from UI and UM, those at UMCU achieved highest pre-module scores in all subscales. The average (SD) score of UMCU students compared to UI and UM in knowledge are 5.04 (0.4) vs. 4.73 (0.69) and 4.24 (0.74); *p* ANOVA<0.001; average (SD) attitude score 4.52 (0.64) vs. 3.85 (0.68) and 3.55 (0.63); *p* ANOVA<0.001; average (SD) behavior score 2.62 (0.55) vs. 2.35 (0.71) and 2.39 (0.92); *p* ANOVA=0.016; average (SD) future use of EBP score 4.32 (0.59) vs. 4.08 (0.62) and 3.7 (0.71); *p* ANOVA<0.001.

**Table 2:** Comparison of scores for subscales for knowledge, attitudes, application and future use of evidence-based practice (EBP), prior to the EBP-modules in the Universitas Indonesia (UI), University of Malaya (UM), and University Medical Center Utrecht (UMCU)

	Mean score (SD)			
		
Factors	UMCU (*n* =100)	UI (*n* =224)	UM (*n* =202)	*p*
EBP knowledge	5.04 (0.40)	4.73 (0.69)	4.24 (0.74)	<0.001 (overall)
				<0.001 (UI vs. UMCU)
				<0.001 (UM vs. UMCU)
				<0.001 (UI vs. UM)
Attitudes toward EBP	4.52 (0.64)	3.85 (0.68)	3.55 (0.63)	<0.001 (overall)
				<0.001 (UI vs. UMCU)
				<0.001 (UM vs. UMCU)
				<0.001 (UI vs. UM)
Personal application and use (behavior) of EBP	2.62 (0.55)	2.35 (0.71)	2.39 (0.92)	0.016 (overall)
				0.016 (UI vs. UMCU)
				0.048 (UM vs. UMCU)
				1.00 (UI vs. UM)
Future use of EBP	4.32 (0.59)	4.08 (0.62)	3.70 (0.71)	<0.001 (overall)
				0.006 (UI vs. UMCU)
				<0.001 (UM vs. UMCU)
				<0.001 (UI vs. UM)

Knowledge, attitude, application, and future use were measured by 6-point Likert scale questions.

The score for each factor was obtained by calculating the mean score of all the items in each respective factor.

*p*-Values were obtained using one-way ANOVA test with post hoc analysis accounting for school.

Table 3 displays the post-module knowledge and attitude scores for the knowledge and attitude subscales. The three medical schools differ, and the UMCU students reached significantly higher scores compared to UI and UM in knowledge (average [SD] of 5.35 [0.51] vs. 4.73 [0.75] and 4.53 [0.72]; *p* of ANOVA<0.001) and in attitude (average [SD] of 4.42 [0.58] vs. 3.83 [0.85] and 3.58 [0.72]; *p* of ANOVA<0.001). The post-module scores of the knowledge subscale were significantly increased (*p* of paired *t*-test<0.001) in UMCU (from average [SD] of 5.04 [0.4] to 5.35 [0.51]) and UM (average [SD] of 4.24 [0.74] to 4.53 [0.72]), but not in UI students. The post-module scores for the attitude subscale did not change in the three medical schools (*p* of paired *t*-test > 0.1).

**Table 3:** Change of scores for subscales for knowledge, attitudes, application, and future use of evidence-based practice (EBP), prior to the EBP-modules in Universitas Indonesia (UI), University of Malaya (UM), and University Medical Center Utrecht (UMCU)

	Mean score (SD)		
		
Factors	Pre	Post	*p*
EBP knowledge			
UMCU	5.04 (0.40)	5.35 (0.51)	<0.001
UI	4.73 (0.69)	4.73 (0.75)	0.096
UM	4.24 (0.74)	4.53 (0.72)	<0.001
Attitudes toward EBP			
UMCU	4.52 (0.64)	4.42 (0.58)	0.136
UI	3.85 (0.68)	3.83 (0.85)	0.586
UM	3.55 (0.63)	3.58 (0.72)	0.470

Knowledge, attitude, application, and future use were measured by 6-point Likert scale questions.

The score for each factor was obtained by calculating the mean score of all the items in each respective factor.

*p*-Values were obtained using paired *t*-test.

## Discussion

Our data on cross-cultural comparison of the short-term effects of EBM teaching in three different medical schools in Asia and Europe shows direct changes in knowledge. Due to the short follow-up, changes in attitude and behavior remain to be seen.

### Questionnaire consistency

To be able to compare the impact of an EBP teaching in three different schools, a validated instrument is needed which corresponds with the objectives and learning goals of the curriculum. Among 104 unique EBP assessment tools identified in one systematic review, only a few of the instruments included in that review evaluate the attitude while none met the pre-determined validity criteria (9). This KAB questionnaire was selected because it measures several components simultaneously. It has been developed and is already implemented in Asian medical schools. The questionnaire was developed through a four-step approach to confirm that the questionnaire has adequate face, content, criterion, and construct validity (7, 10). Johnston et al. (7) found that the Cronbach's α values of each component in the original questionnaire ranged from 0.71 to 0.88. As we slightly adapted the questionnaire, we repeated the analysis for the internal consistency of the questionnaire. In this study, we observed Cronbach's α in the same range.

### Limitations of study

This study has several limitations, which may complicate the interpretation of findings. First, the use of English as the original language of the questionnaire could cause difficulties for some students especially those from Indonesia and Netherlands. The second limitation is differences in some baseline characteristics such as the year that the medical student is currently in and also previous exposure to EBM due to differences in curriculum structure as well as cultural and environmental aspects in each country. Cultural and linguistic differences as well as characteristic differences might cause bias to the results due to potential differences in the perception and interpretation of terms (11, 12). Third, this questionnaire measured self-reported outcomes, which could potentially lead to systematic selection of socially desirable responses.

### KAB comparison among medical schools

We found significant differences in all components measured among students in the three medical schools before the module was implemented. This could be due to differences in local conditions, that is, curriculum structure and student years of education.

In UMCU, the CE and the EBM are taught in separate modules. The CE is taught in the 3rd year while the EBM in the 6th year. In UI, the CE-EBM module is given in blocks as a 4-week module. However, students had also already learned about research methodology and biostatistics during their 1st year. In UM, the CE-EBM is taught twice a week within the Social Preventive Medicine module, which is conducted over 3 months. The fact that the UMCU students achieved the highest baseline score in all components compared to UI and UM shows that their previous CE module might have significantly contributed to their KAB toward EBP.

The UI students scored higher than the UM students in knowledge, attitudes, and future use of EBM, which could also be related to previous exposure during research module in UI and the difference in students education years (4th year in UI vs. 3rd year in UM). All the behavior questions asked about how frequent the students accessed different kinds of medical evidence such as text books, Medline, and Cochrane database. Even though access to medical literature in UM is generally better than UI, similar behavior was found among the students in the two medical schools.

Similar conditions were still observed in the three medical schools after the module was implemented – the knowledge and attitude score of the UMCU students are still higher compared to the two Asian schools. Even though statistically significant changes in EBP knowledge of UMCU and UM students were found after the module, the actual effect size was small. CE and EBM modules in UMCU have been implemented for 7 years since revision of the curriculum. Even with higher pre-module scores, EBM teaching still adds knowledge to the UMCU students, however small.

Cultural differences in responding to the 6-point Likert scale format of the questionnaire, which required the students to choose between strongly disagree to strongly agree, could also influence the result. Further analysis of each scale selection revealed that the UI and UM students were more likely to select the middle point (agree or disagree) when expressing their opinions compared to their Dutch colleagues who were more bold and did not hesitate to express stronger opinions. This is similar to other studies that compared American students with Asian students (Chinese and Japanese) (13, (14). The Asian students were more likely than the Americans to use the midpoint on the scales. However, students’ uncertainty about their answer could also contribute to the selection of the midpoint.

The similarity between the scores achieved by the UI and UM students, as observed by Johnston et al. (7) at Hong Kong medical school confirms our view. Moreover, only an increase in EBP knowledge was observed in both studies. Nevertheless, a more recent study also by Johnston (15) reported that attitude and behavior could also increase after the module, and Cheng et al. (10) in National Taiwan University Taipei found improvement in all components after the module. Post-module assessment in our study was conducted on the final day of the module, which was run as a stand-alone module (in UI and UMCU) or integrated with SPM module (in UM) as opposed to being incorporated with the clinical rotation as in the studies by Johnston (15) and Cheng (10). Because of that, it is not sufficient to observe changes in attitude as well as application or behavior.

### Future application of the KAB questionnaire

The questionnaire includes different aspects on knowledge, attitudes, behavior, and future use of EBP principles as described in Sicily statement (1). These aspects are suggested as dimensions for evaluation of EBP learning in the CREATE framework (9). Still, the questionnaire does not sufficiently reflect all the EBP principles of asking, searching, appraising, integrating, and evaluating separately.

Our data show that this KAB questionnaire is helpful in providing information on the differences in knowledge, attitude, behavior, and future use of EBP in three different medical schools in Asia and Europe prior to EBP teaching. Moreover, it has been shown to pick up changes in the knowledge of students, even with higher pre-module scores. This KAB questionnaire can perhaps be used as an instrument to facilitate reflection of KAB for EBP. However, cultural aspects might influence the response, and the changes in the attitude and behavior could not be observed immediately at the end of the EBP teaching (so no direct effects on these two dimensions).

## Conclusion

We show that EBP teaching has direct short-term effects on knowledge but not on attitude. The validated KAB questionnaire is very useful and helpful in evaluating the effects of EBP teaching. It has managed to provide information on the pre-EBP module differences in knowledge, attitude, behavior, and future use of EBP in three different medical schools in Asia and Europe, and on the post-EBP module change in knowledge.

## References

[1] Dawes M, Summerskill W, Glasziou P, Cartabellotta A, Martin J, Hopayian K (2005). Sicily statement of evidence-based practice. BMC Med Educ.

[2] Crilly M, Glasziou P, Heneghan C, Meats E, Burls A (2009). Does the current version of ‘Tomorrow's Doctors’ adequately support the role of evidence-based medicine in the undergraduate curriculum?. Med Teach.

[3] Green M (2001). Identifying, appraising, and implementing medical education curricula: a guide for medical educators. Ann Intern Med.

[4] Straus SE, Green ML, Bell DS, Badgett R, Davis D, Gerrity M (2004). Evaluating the teaching of evidence based medicine: conceptual framework. BMJ.

[5] Widyahening IS, van der Heijden GJMG, Moy Foong Ming, van der Graaf Y, Sastroasmoro S, Bulgiba A (2012). From West to East; experience with adapting a curriculum in evidence-based medicine. Perspect Med Educ.

[6] Rovers MM, van der Heijden GJMG (2010). Translating research evidence into action in daily practice. Otolaryngol Head Neck Surg.

[7] Johnston JM, Leung GM, Fielding R, Tin KYK, Ho LM (2003). The development and validation of a knowledge, attitude and behaviour questionnaire to assess undergraduate evidence-based practice teaching and learning. Med Educ.

[8] Bland JM, Altman DG (1997). Cronbach's alpha. BMJ.

[9] Tilson J, Kaplan S, Harris J, Hutchinson A, Dragan I, Niederman R (2011). Sicily statement on classification and development of evidence-based practice learning assessment tools. BMC Med Educ.

[10] Cheng HM, Guo FR, Hsu TF, Chuang SY, Yen HT, Lee FY (2012). Two strategies to intensify evidence-based medicine education of undergraduate students: a randomised controlled trial. Ann Acad Med Singapore.

[11] Hilton A, Skrutkowski M (2002). Translating instruments into other languages: Development and testing processes. Cancer Nursing.

[12] Sperber A (2004). Translation and validation of study instruments for cross-cultural research. Gastroenterology.

[13] Lee JW, Jones PS, Mineyama Y, Zhang XE (2002). Cultural differences in responses to a Likert scale. Res Nurs Health.

[14] Chen C, Lee S-Y, Stevenson HW (1995). Response style and cross-cultural comparisons of rating scales among East Asian and North American students. Psychol Sci.

[15] Johnston JM, Schooling CM, Leung GM (2009). A randomised-controlled trial of two educational modes for undergraduate evidence-based medicine learning in Asia. BMC Med Educ.

